# Factors associated with prolonged use of newly prescribed gabapentinoids in older adults undergoing colorectal surgery

**DOI:** 10.1186/s13741-026-00714-0

**Published:** 2026-07-02

**Authors:** Sohil Patel, Karen Trang, Tasce Bongiovanni, Siqi Gan, John Boscardin, Elizabeth C. Wick

**Affiliations:** 1 School of Medicine, University of California San Francisco School of Medicine, San Francisco, CA USA; 2 Department of Surgery, University of California San Francisco, 513 Parnassus Ave, HSW #1611, San Francisco, CA 94143 USA; 3 Northern California Institute for Research and Education, San Francisco, CA USA; 4 Division of Geriatrics, University of California San Francisco, San Francisco, CA USA; 5 Department of Medicine, University of California San Francisco School of Medicine, San Francisco, CA USA

**Keywords:** Gabapentinoids, Colorectal surgery, Older adult, ERAS, Opioid use

## Abstract

**Background:**

Gabapentinoids are one component of multimodal analgesia in enhanced recovery programs after colorectal surgery. Long-term use can be especially harmful in older adults. This study sought to identify factors associated with prolonged gabapentinoid use in older adults following colorectal surgery.

**Methods:**

This retrospective cohort study analyzed data on a 20% representative sample of patients with Medicare. Patients were included if they were ≥ 66 years old, underwent colorectal surgery between October 1, 2015 and June 30, 2018, had continuous Part D enrollment during the time of operation, and were gabapentinoid naïve. Patients were excluded if they died within 180 days after surgery or were discharged to hospice or skilled nursing facility. The main outcome measure was prolonged gabapentinoid use, defined as prescription refills > 90 days after discharge, in those newly prescribed a gabapentinoid.

**Results:**

Among the 979 patients who met inclusion criteria and had a new gabapentinoid prescription after colorectal surgery, 22% had prolonged gabapentinoid use. Concurrent peri-discharge opioid use was associated with reduced likelihood of prolonged gabapentinoid use (OR 0.48 [0.34, 0.66], *p* < 0.0001). In patients who had both opioid and gabapentinoid prescriptions at discharge, opioid use beyond 90 days was associated with prolonged gabapentinoid use (OR 3.45 [2.43, 4.89], *p* < 0.0001). Patients discharged home with home health services had increased likelihood of prolonged gabapentinoid use (OR 1.53 [1.08, 2.17], *p* = 0.047).

**Conclusions:**

Factors associated with prolonged gabapentinoid use after colorectal surgery in the older adult population include initial prescription of a gabapentinoid without opioids, prolonged opioid use beyond discharge, and discharge to home with health services. Identifying these factors may reveal potentially modifiable interventions to curb prolonged gabapentinoid use in this population.

## Introduction

Enhanced recovery after surgery (ERAS) pathways, guideline-based protocols for perioperative care, are commonly used strategies to optimize outcomes after colorectal surgery. Successful implementation of ERAS is associated with shorter lengths of stay, reduction in surgical complications, and improved patient experience. One key component of ERAS is a multimodal pain regimen, which may synergistically reduce perioperative pain while minimizing the risk of adverse medication effects (Decker et al. 2021). More specifically, many clinicians are increasingly prescribing non-opioid pain medications in an effort to reduce opioid consumption (Wick et al. 2017).

Professional societies have especially popularized the use of gabapentinoids (pregabalin and gabapentin) for reducing pain in the acute perioperative setting (Practice Guidelines for Acute Pain Management 2012). This advocacy was based on initial studies showing that preoperative use of gabapentinoids may reduce opioid consumption in the first 24 h following surgery (Chamberlain et al. 2016; Gedda et al. 2023; Helander et al. 2017; Mujukian et al. 2020; Sarac et al. 2022; Froehlich et al. 2024; Mishra et al. 2016). However, these findings were limited by their high risk of bias and low sample sizes (Fabritius et al. 2017 Fabritius et al. 2017). More recent studies have shown only a small or no clinically meaningful benefit of gabapentinoids for perioperative pain management (Fabritius et al. 2016; Verret et al. 2020; Hah et al. 2018). Furthermore, gabapentinoids are known to be associated with adverse effects, such as dizziness, drowsiness, confusion, and falls (Verret et al. 2020; Kumar and Habib 2019; Park et al. 2022). These effects are even more pronounced with higher doses, prolonged use, concomitant opioid use, and in the older adult population (age ≥ 65 years). (American Geriatrics Society 2019)

The older adult population is particularly vulnerable to the adverse effects of gabapentinoids for various reasons, including decreased renal clearance in the setting of polypharmacy, thus increasing the risk for drug-drug interactions (McKeown 2015). Despite this, older adults make up a higher proportion of gabapentinoid users than younger adults (age < 65 years) (Johansen 2018). In the postoperative setting, older adults may be prescribed gabapentinoids as an adjunct for pain control. This is especially concerning because when gabapentinoids are prescribed along with opioids, the two medications can increase the risk of serious adverse effects such as respiratory depression, worsened neurocognitive function, and increased mortality (Bykov et al. 2020; Corriere et al. 2023). Additionally, more than one-fifth of older adults who are prescribed gabapentinoids after surgery, likely as part of multimodal pain regimens, remain on the medication beyond 90 days after discharge, indicating continued use past the postoperative period. (Bongiovanni et al. 2022)

Although older adult patients are likely being prescribed gabapentinoids as a part of ERAS protocols after colorectal surgery, there is a relative paucity of information about persistence of these prescriptions. This is particularly important to understand in colorectal surgery, as it is an area with broad adoption of multimodal analgesia. We therefore sought to identify the proportion of older adults being prescribed gabapentinoids upon discharge following colorectal surgery and factors associated with prolonged use of gabapentinoids beyond the perioperative period (> 90 days). Understanding these trends may identify modifiable interventions to improve quality of care and safety for patients prescribed multimodal pain medications after surgery.

## Materials

### Study population

The study population is a 20% representative sample of patients with Medicare, obtaining information from MedPAR file. We used the Master Beneficiary Summary File (MBSF) with the additional segments “chronic conditions,” “additional chronic conditions,” and National Death Index (NDI) to extract information about age, gender, race, and discharge location. Prescription fills were extracted from Part D file. Medicare Part D is the federal outpatient prescription drug benefit through which the prescription fills analyzed in this study were captured. This study was approved by the University of California, San Francisco Institutional Review Board.

We identified patients who underwent colorectal surgery between October 1, 2015, to June 30, 2018, using the 2023 ICD-10-PCS codes under the “COLO and “REC” from the National Healthcare Safety Network (NHSN). Patients were included if they were ≥ 66 years old at the time of the operation, had continuous Part D enrollment for at least 90 days before and 180 days after the procedure, and had at least one prescription filled 90 days prior to the procedure. This ensured that the patients were actively using their Medicare Part D plan. Exclusion criteria were death within 180 days after surgery or discharge to hospice or a skilled nursing facility. If a patient had multiple colorectal surgeries, we selected the latest procedure date.

Finally, to analyze patients with prolonged gabapentinoid refills, we restricted our analysis solely to patients with *new* gabapentinoid use following colorectal surgery. *New* gabapentinoid use was defined as prescription of a gabapentinoid 7 days before or after the procedure date, or discharge date for inpatient stay. We excluded patients who were not gabapentinoid naïve, i.e. used gabapentinoids for up to 90 days prior to surgery. (Thiels et al. 2019)

### Outcome and analyses

Our primary outcome was *prolonged* gabapentinoid use, defined as prescription refills 90–180 days after discharge. This time period was defined based on prior studies of opioid use after surgeries (Thiels et al. 2019; Brummett et al. 2017). Concurrent opioid use was analyzed at two timepoints: 1) fills of opioid 7 days before admission or after the discharge date, and 2) for those prescribed opioids at peri-discharge, refills 90–180 days after discharge. Disposition location was categorized as home or home with organized health service.

Data are reported as mean ± standard deviation (SD), median ± interquartile range (IQR), or counts with percentages. Student’s t-test was used to compare continuous variables, and chi-square tests to compare categorical variables. Multivariable logistic regression was used to identify risk factors for prolonged use of gabapentinoids beyond 90 days after discharge. Risk factors assessed were age, gender, race, length of stay, concurrent opioid use, and disposition. A *p-*value of < 0.05 was considered significant. All analyses were performed in SAS (Version 9.4, SAS Institute, Cary, NC).

## Results

### Patient demographics

We identified 979 patients who underwent colorectal surgery and met inclusion/exclusion criteria, including a new prescription for gabapentinoid at discharge (Table 1). The mean age was 73.7 ± 5.9 years, 60% of patients were female, and 81% were White. Sixty-two percent of patients had a concurrent opioid prescription in the peri-discharge setting, and 23% of patients refilled opioids beyond the postoperative period (90–180 days post-discharge). The median length of stay (LOS) was 4 days (IQR 3–7 days). Most patients were discharged either home (66%) or home with home health services (34%). The most common surgical procedures in this cohort were open right hemicolectomy (14.0%), open sigmoid colectomy (12.9%), laparoscopic right hemicolectomy (12.7%), and laparoscopic sigmoid colectomy (6.0%).

**Table 1:** Univariate associations with prolonged gabapentinoid use (> 90 days after discharge)

	Total Prescribed Gabapentinoids at Discharge^1^ (N = 979)	No Prolonged Gabapentinoid Use^2^ (N = 768)	Prolonged Gabapentinoid Use^2^ (N = 211)	*P* value
Age				
Mean (SD)	73.7 (5.9)	73.7 (5.9)	73.5 (5.7)	0.65
Gender				0.63
Female	585 (59.8%)	462 (60.2%)	123 (58.3%)	
Male	394 (40.3%)	306 (39.8%)	88 (41.7%)	
Race				0.22
White	791 (80.8%)	612 (79.7%)	179 (84.8%)	
Black	79 (8.1%)	63 (8.2%)	16 (7.6%)	
Hispanic	62 (6.3%)	51 (6.6%)	11 (5.2%)	
Other	47 (4.8%)	42 (5.5%)	5 (2.4%)	
Concurrent Opioid Prescription				
Peri-discharge				0.0003
No	369 (37.7%)	267 (34.8%)	102 (48.3%)	
Yes	610 (62.3%)	501 (65.2%)	109 (51.7%)	
Prolonged opioid use (90–180 days) after discharge				< 0.0001
No	753 (76.9%)	632 (82.3%)	121 (57.3%)	
Yes	226 (23.1%)	136 (17.7%)	90 (42.7%)	
Length of Stay				
Median (Q1, Q3)	4 (3, 7)	4 (3, 7)	6 (3, 10)	0.0001
Disposition Location				< 0.0001
Home	641 (65.5%)	526 (68.5%)	115 (54.5%)	
Home Health Service	333 (34.1%)	238 (31.0%)	95 (45.0%)	

*SD* standard deviation, *Q1* first quartile, *Q3* third quartile

^1^Percentages in this column are out of total prescribed gabapentinoids at discharge (*N* = 979)

^2^Percentages in these columns are out of their respective categories (i.e. no prolonged or prolonged gabapentinoid use)

Among our cohort of 979 patients, 22% had prolonged gabapentinoid use. Baseline age, gender, and race were not significantly different between those who had prolonged use versus those who did not. However, concurrent peri-discharge prescription of opioids was less common in patients with prolonged gabapentinoid use (51.7% vs 65.2%, p = 0.0003). In patients who were prescribed opioids at discharge, prolonged opioid refills (90–180 days) after discharge were significantly more common in those with prolonged gabapentinoid use than those without (42.7% vs 17.7%, p < 0.0001). Patients with prolonged gabapentinoid use also had longer hospital LOS with a median of 6 (IQR 3–10 days) versus 4 days (IQR 3–7 days) in those who did not have prolonged gabapentinoid use (p = 0.0001). Finally, discharge with home health services was more common in those with prolonged gabapentinoid use (45.0% vs 31.0%, p < 0.0001).

### Multivariable analysis

Several variables (age, gender, race, LOS, concurrent opioid use, and disposition) were used in a multivariate logistic regression model to identify which were associated with prolonged gabapentinoid use (Table 2). Concurrent peri-discharge use of opioids decreased the likelihood of prolonged gabapentinoid use (OR 0.48 [0.34, 0.66], p < 0.001). However, in patients who had both opioid and gabapentinoid prescriptions at discharge, opioid use beyond 90 days was associated with prolonged gabapentinoid use (OR 3.45 [2.43, 4.89], p < 0.0001). Patients who were discharged home with home health services had increased likelihood of prolonged gabapentinoid use when compared to those discharged home without home care (OR 1.53 [1.08, 2.17], p = 0.047).

**Table 2:** Multivariable logistic regression model for characteristics associated with prolonged gabapentinoid use (> 90 days after discharge)

	**OR (95% CI)**	***P* Value**
Age	0.99 (0.96, 1.02)	0.50
Gender		0.43
Male	ref	
Female	0.88 (0.63, 1.22)	
Race		0.23
White	ref	
Black	0.84 (0.45, 1.56)	
Hispanic	0.74 (0.37, 1.49)	
Other	0.38 (0.15, 1.01)	
Concurrent Opioid Prescription		
Peri-discharge		< 0.0001
No	ref	
Yes	0.48 (0.34, 0.66)	
90–180 days after discharge		< 0.0001
No	ref	
Yes	3.45 (2.43, 4.89)	
Length of Stay	1.02 (1.00, 1.04)	0.06
Disposition Location		0.047
Home	ref	
Home Health Service	1.53 (1.08, 2.17)	

*OR* odds ratio (reference = no prolonged use), *ref.* reference

## Discussion

In this study, we identified risk factors for prolonged gabapentinoid use in older adults after colorectal surgery. We found that patients who were prescribed gabapentinoid without opioids in the perioperative setting were more likely to use gabapentinoids beyond the postoperative period. However, if patients were prescribed both, those with prolonged use of opioids were more likely to also continue using gabapentinoids in a prolonged fashion. Finally, patients who were discharged home with home care services were more likely to continue using gabapentinoids than those who were discharged without home care services. Based on these findings, it appears that there is an opportunity to improve education around the benefits of perioperative gabapentinoids, particularly the time-limited benefits.

Although gabapentinoids have been widely adopted as opioid-sparing adjuncts within ERAS pathways, an understanding of their pharmacology would be helpful for explicating why their appropriate use may be time-limited. Gabapentinoids bind selectively to the α2δ auxiliary subunit of presynaptic voltage-gated calcium channels, thereby attenuating the release of excitatory neurotransmitters. This mechanism explains both their analgesic and their antiepileptic activity (Sills 2006). This α2δ-mediated central effect has also been used to explain their utility against neuropathic and acute postoperative pain as well as their contribution to reducing perioperative opioid requirements. However, because recent trials have indicated only a small or non–clinically-meaningful analgesic benefit in the acute perioperative setting, (Fabritius et al. 2016; Verret et al. 2020; Hah et al. 2018) the pharmacologic rationale for continuing a gabapentinoid well beyond the immediate postoperative period is limited, and prolonged α2δ-mediated exposure may instead contribute to the dose- and duration-dependent adverse effects that are of particular concern in older adults.

Three percent of older adult patients in our cohort received a new prescription for a gabapentinoid following colorectal surgery, which is consistent with the reported usage of gabapentinoids in the community based on current literature. Although gabapentinoid use in the United States rose from 1.2% in 2002 to 3.9% in 2015, (Johansen 2018) it has continued to increase since then, with the most recent national estimate reaching 4.7% in 2021 (Johansen and Maust 2024). Importantly, the older adult population is particularly susceptible to polypharmacy and medication inertia, and intentional deprescribing practices have previously been shown to reduce inappropriate medication use (Martin et al. 2018). Therefore, identifying patients likely to have prolonged gabapentinoid use would allow for more targeted interventions to minimize risks.

With regards to prolonged opioid use after colorectal surgery, it is noteworthy that the 23.1% rate of prolonged opioid use observed in our cohort appears higher than the 6% to 16% range described in other analyses of new persistent opioid use after surgery (Brummett et al. 2017; McKie et al. 2025). Notably, our cohort was restricted to older adults aged > 65 years in Medicare who were newly prescribed a gabapentinoid, a subgroup that is, by selection, predisposed to a greater perioperative pain burden and the need for more intensive multimodal regimens than the broader and predominantly younger surgical populations from which the lower estimates were derived. Furthermore, colorectal resection constitutes major abdominal surgery, for which persistent postoperative opioid use has consistently been reported toward the higher end of the surgical range.

To our knowledge, no prior study has characterized prolonged gabapentinoid use specifically after colorectal surgery. However, our results are consistent with findings from a previous study conducted on the general post-surgical population, which showed that opioid use at > 90 days after discharge was strongly associated with prolonged gabapentinoid use after surgery, particularly after emergency surgeries (Bongiovanni et al. 2022). Therefore, even though clinicians may prescribe gabapentinoids in an effort to limit opioid consumption, in settings in which the two agents are used together the gabapentinoid may ultimately be continued for longer. Patients receiving opioids may be more likely to continue gabapentinoids for several reasons, including the pursuit of synergistic analgesia, attempts to reduce opioid consumption, and the management of opioid withdrawal symptoms. However, the prolonged concurrent use of both agents is of particular concern in older adults because concomitant exposure may amplify adverse effects such as sedation, dizziness, and respiratory depression. Although our administrative dataset was not designed to capture such events directly, the harms associated with gabapentinoid exposure in older adults are well documented in the literature. A 2021 Medicare cohort study indicated that co-prescription of an opioid and a gabapentinoid was associated with an increased risk of falls (adjusted hazard ratio 1.18 [1.02, 1.37]) relative to other psychotropic combinations and a 2025 geriatric review similarly reported increased risks of falls and fractures, respiratory depression, and cognitive and functional impairment with gabapentinoids in older patients (Gahr 2026; Shah et al. 2021). Furthermore, perioperative gabapentin use has previously been associated with in-hospital adverse clinical events, including delirium, among older adults after major surgery (Park et al. 2022). Despite some evidence indicating a risk of addiction with gabapentinoid use, (Evoy et al. 2021) as well as reports of misuse (Buttram and Kurtz 2021), gabapentinoid is not classified as a scheduled substance at the federal level. However, state legislatures and jurisdictions are increasingly adopting guidelines in an effort to thwart widespread consumption of gabapentinoids. (Campbell et al. 2021)

We were initially surprised to find that prescribing a gabapentinoid without a concurrent opioid was associated with prolonged gabapentinoid use, and although the reason for this association remains unclear, several explanations are plausible. One possibility is inadequate patient and provider education regarding the time-limited perioperative role of gabapentinoids. Additionally, patients’ perceptions of medications have been shown to influence adherence, which could lead to inappropriate use of pain medication (Edworthy and Devins 1999). Because gabapentinoids are frequently prescribed off-label for pain, patients may be less familiar with the indication for their prescription than they are for more commonly prescribed analgesics such as opioids. Consequently, when a gabapentinoid is prescribed without another pain medication, patients may not realize that it was intended for short-term postoperative pain relief only and may consume it longer than the indicated period. Although most patients would not be expected to experience significant pain 90 days after surgery, chronic postsurgical pain is not uncommon after colorectal surgery. A 2021 prospective study reported chronic postsurgical pain in 32.1% of patients at 3 months and 21.8% at 6 months, (Jin et al. 2021) and a 2022 matched analysis of visceral surgery further indicated that preoperative pregabalin exposure was protective against the development of chronic postsurgical pain (Perrodin et al. 2022). Consequently, a subset of patients with prolonged gabapentinoid use may have legitimate ongoing neuropathic or chronic pain for which the gabapentinoid would be helpful, although our data do not permit us to distinguish between these possibilities.

It is less clear why patients discharged home with home health services were more likely to continue gabapentinoids than those discharged home without such services. We note that in the United States, a home health service refers to skilled nursing including wound care, physical/occupational therapy, or other professional care delivered in a patient’s own residence after discharge. Because home health services are typically ordered for patients with greater functional dependency upon discharge, the need for home health services may serve as a surrogate for unmeasured illness severity. Therefore, the association we observed may in part reflect increased postoperative pain or greater disease burden related to the intensity of the surgery. Furthermore, given that chronic postsurgical pain after colorectal surgery has been reported in roughly one-fifth to one-third of patients, (Jin et al. 2021) and that preoperative gabapentinoid has been reported to be protective against such pain (Perrodin et al. 2022), a meaningful minority of patients discharged with home health services may have a genuine ongoing indication for continued gabapentinoid use rather than inappropriate continuation. Distinguishing a valid ongoing indication from inappropriate continuation remains an important question for future studies.

The factors we identified operate at the level of prescribing behavior and the care pathway rather than as direct markers of pain severity, and this characteristic is precisely what renders them modifiable targets for quality improvement. Thus, we propose several strategies to deprescribe gabapentinoids as part of the perioperative care pathway on at least three levels. At the hospital level prior to discharge, embedding an explicit stop date and a documented indication within the discharge gabapentinoid order and/or summary may help signal the intended short-term use of the prescription. For the transitional period (the period after discharge but before follow-up visit), multidisciplinary transitional pain services have been shown to successfully accelerate postoperative opioid dose reduction (Ladha et al. 2024). Such services may be similarly applied to reducing postoperative usage of gabapentinoids. At the primary care level, a deliberate review of perioperatively initiated gabapentinoids at a routine follow-up visit may reduce inappropriate prolonged use (Growdon et al. 2024). Most importantly, these proposals may be effective when framed as an extension of opioid stewardship, given that persistent opioid use after surgery declined from 2.7% to 1.1% over the last decade with dedicated stewardship efforts. (Luby et al. 2025)

### Limitations

Our study has several limitations, some of which have been described elsewhere in a related study conducted by our team (Bongiovanni et al. 2022). First, gabapentin and pregabalin were analyzed together as a single gabapentinoid class, although the two agents differ meaningfully in their absorption and oral bioavailability. However, the aim of this study concerns prescribing behavior rather than the comparative pharmacology of the two agents, and notably the two agents are routinely treated as a single class in most recent colorectal surgery guidelines (American Geriatrics Society 2019; Irani et al. 2023). Second, although length of stay was included as a covariate, it is an imperfect surrogate for surgical complexity and overall illness burden, and our analysis did not capture procedure acuity (elective versus emergent) or disease type (benign versus malignant). Consequently, our association may be susceptible to residual confounding. Third, although the cohort was drawn from a nationally representative 20% Medicare sample, the limited racial and ethnic diversity of the population leaves the study underpowered to detect race-specific associations with prolonged use. Furthermore, certain racial and ethnic minority groups may be misclassified within Medicare administrative data. (Jarrín et al. 2020) Fourth, restricting the analysis to patients newly prescribed a gabapentinoid may limit the generalizability of our findings to this prescribing subgroup and may introduce selection bias. However, the selection of this sample was a deliberate design choice to isolate patients who initiated gabapentinoid specifically after surgery. Finally, the Part D benefit may not capture all medication prescriptions, particularly when only a short supply was dispensed or when medications were supplied directly from the hospital at discharge, and prescription fills or refills may not necessarily mirror patients’ actual use of medications.

## Conclusion

Although gabapentinoid use may be beneficial for postoperative pain management as outlined in ERAS pathways, more than a fifth of older adult patients prescribed a gabapentinoid are still using this medication long after colorectal surgery. We identified several factors associated with prolonged gabapentinoid use after colorectal surgery in the older adult population, including initial prescription of a gabapentinoid without opioids, prolonged opioid use beyond discharge, and discharge to home with health services. Further prospective studies are needed to understand the rationale behind prolonged use and to potentially design improvement efforts to target these modifiable risk factors.

## Data Availability

The datasets analyzed during the current study are available in the MedPAR and MBSF files with the following links: https://resdac.org/cms-data/files/medpar, https://resdac.org/cms-data/files/mbsf-base

## Abbreviations

ERAS: Enhanced recovery after surgery
MBSF: Master Beneficiary Summary File
NDI: National Death Index
NHSN: National Healthcare Safety Network
SD: Standard deviation
IQR: Interquartile range
LOS: Length of stay
OR: Odds ratio
